# Zn_1−x_Te_x_ Ovonic Threshold Switching Device Performance and its Correlation to Material Parameters

**DOI:** 10.1038/s41598-018-30207-0

**Published:** 2018-08-07

**Authors:** Yunmo Koo, Hyunsang Hwang

**Affiliations:** Department of Materials Science and Engineering, Pohang University of Science and Technology (POSTECH), Pohang, 790-784 Republic of Korea

## Abstract

We have experimentally demonstrated a strong correlation between the electrical properties of Zn_1−x_Te_x_ Ovonic threshold switching (OTS) selector device and the material properties analysed by X-ray diffraction (XRD), spectroscopic ellipsometry, and X-ray photoelectron spectroscopy (XPS). The correlation and the key material parameters determining the device performances were investigated. By comparing the experimental data with the calculation results from various analytical models previously developed for OTS materials, the electrical properties of the device were shown to be dependent on the key material parameters; the concentration of sub-gap trap states and the bandgap energy of the OTS material. This study also experimentally demonstrated that those key parameters have determined the device performance as expected from the analytical model. The origin of the OTS phenomenon and conduction mechanism were explained both experimentally and theoretically. This leads to better understanding of the conduction mechanism of OTS devices, and an insight for process improvement to optimize device performance for selector application.

## Introduction

The density and performance of memory devices are the top priority concerns in the memory industry^1–3^. To maximize both density and performance of memory devices, the high-density X-point memory array structure that consists of 4F^2^ memory devices with 2-terminal access devices, known as *selector* devices, has been widely investigated^4–8^. In this structure, high density can be achieved by closely packing high-performance memory devices in number of word lines and bit lines. In such an array, selector devices are necessary for delivering enough operation voltage to the *selected* memory device, while inhibiting leakage current flow from a number of *unselected* memory devices. For this purpose, selector devices are required to have low resistance at higher voltage to operate the *selected* memory devices, while having a high resistance at lower voltage to suppress sneak path leakage current from *unselected* memory devices. Ovonic threshold switching (OTS) materials have been receiving great attention as a promising candidate for selector devices^7–13^. OTS devices are known for their favourable resistive switching which is field-dependent, volatile, instant, abrupt, fast-switching, and repeatable, and thus suitable for selector applications in high-density X-point memory arrays^4,7–14^. The OTS phenomenon has been widely studied theoretically; however, its conduction mechanism and corresponding analytical model is still a highly controversial topic^14–22^. In this study, material characteristics of binary OTS material Zn_1−x_Te_x_ and its device performance were studied, and various analytical models^19–22^ were compared with the experimental data. As a result, the origin of OTS phenomenon and the performance-determining material parameters has been experimentally confirmed.

## Results

### OTS phenomenon in binary material ZnTe

**Figure 1:**
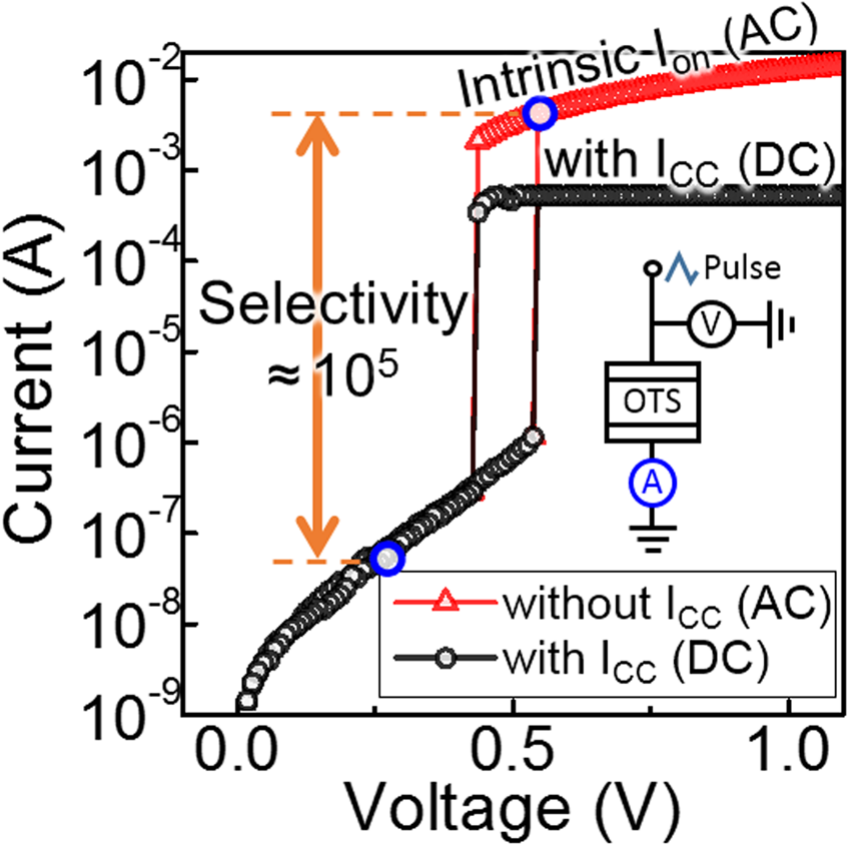
I–V curves of the 2-terminal Zn_0.35_Te_0.65_ device showing exceptional selector performance by OTS phenomenon. The device strongly inhibits leakage current under low voltage (|V_a_| < V_th_) while supplies high current under higher voltage (|V_a_| > V_th_) for operating memory, thus suitable for selector application with its high selectivity of 10^5^. Black dotted line data was measured in a longer time scale (>100 ms, DC) with current compliance, while the intrinsic *on* state conduction shown as red dotted line data was measured in a shorter time scale (<1 ms, AC) to prevent damage to the device.

The 2-terminal ZnTe devices with the experimental details stated in the *Methods* section showed OTS phenomenon when external bias (V_a_) higher than the threshold voltage (V_th_) was applied (Fig. 1). This field-dependent volatile resistance switching behaviour was instant, abrupt, fast, and repeatable. At lower voltage (|V_a_| < V_th_), the high resistance of the device in its *off* state kept the *off* state current (I_off_) remarkably low. At higher voltage (|V_a_| > V_th_), the device undergoes OTS phenomenon and switched to *on* state with low resistance; thus current through the device in the *on* state (I_on_) increased considerably. The volatile *on* state is maintained as long as high voltage is supplied. The optimized ZnTe device showed high selectivity (the ratio of I_on_ at V_th_ to I_off_ at 1/2 V_th_) of 10^5^, which is exceptionally high compared to various types of selector devices^5–8^, and thus promising for selector device application in high-density X-point memory arrays.

### Experimental observations of ZnTe with compositional change

**Figure 2:**
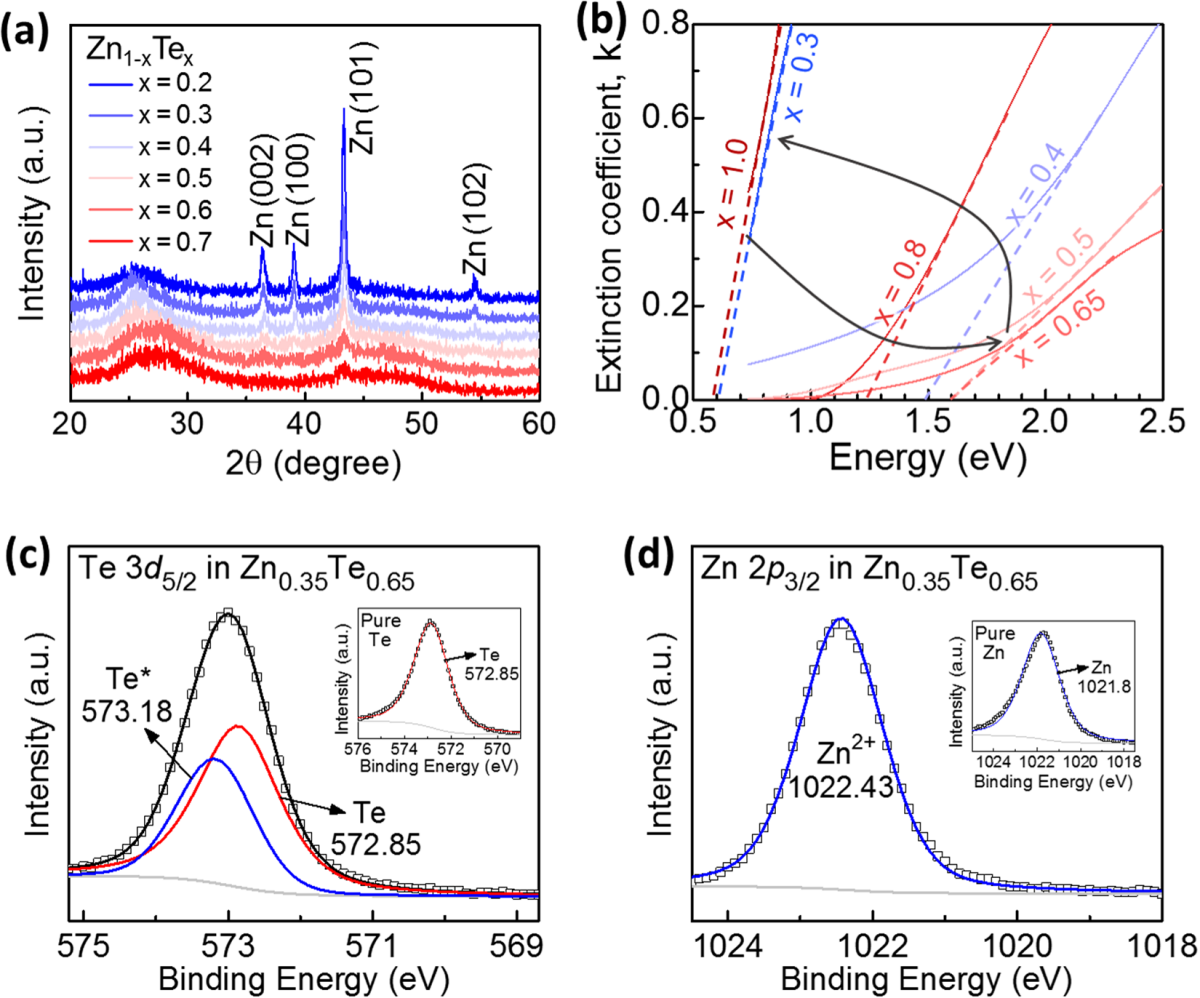
Properties of binary OTS material Zn_1−x_Te_x_ with various composition. (**a**) XRD results showing amorphous phase of Zn_1−x_Te_x_ film sputtered in room temperature. Crystalline phase was detected only in highly Zn-rich composition. (**b**) Spectroscopic ellipsometry results showing bandgap energy of Zn_1−x_Te_x_ according to composition. The arrows represents the gradual change of the optical bandgap according to the composition. The gradual change is strongly coherent with the electrical properties of the devices. XPS spectra of (**c**) Te 3*d*_5/2_ and (**d**) Zn 2*p*_3/2_ core levels measured in Zn_0.35_Te_0.65_ sample. XPS spectra for pure Te and pure Zn were measured for comparison (see figure insets). Large amount of unbound Te lone-pairs was found from Zn_0.35_Te_0.65_.

**Figure 3:**
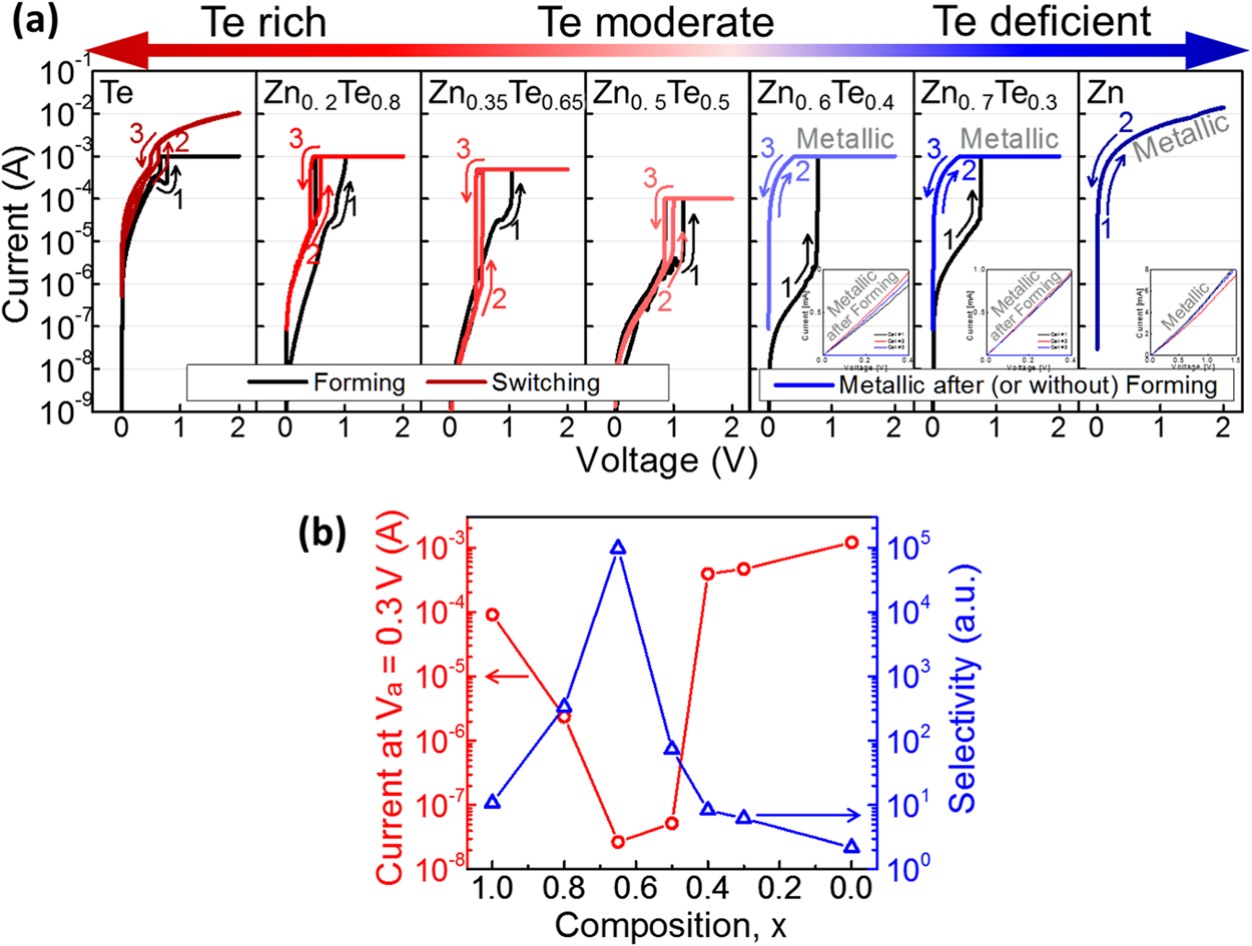
(**a**) Electrical responses of 2-terminal Zn_1−x_Te_x_ devices and (**b**) corresponding selector performances at various composition. The I-V curve of pure Te is the experimental evidence that OTS phenomena of the device is attribute to Te atoms. Mixing proper amount of Zn into Te can dramatically improve selector performance by decreasing I_off_, while over-doped Zn content may cause device performance degradation and even cause metallic failure in Zn-rich composition: the excessive Zn atoms in the device (as shown in Fig. 2(a)) may form Zn filament under bias, serving as conductive path. Optimization of material composition (Zn_0.35_Te_0.65_) results in efficient inhibition of leakage current and higher selectivity, thus suitable for selector application.

Figures 2 and 3 show experimental results of various material properties of binary OTS material Zn_1−x_Te_x_ according to composition. X-ray diffraction (XRD) analysis results are shown in Fig. 2a. XRD peaks representing crystalline phase were only detected in highly Zn-rich samples (0 < x < 0.5), and all the crystalline phases detected were Zn crystals. This shows that in highly Zn-rich samples, the excessive Zn atoms are present in the form of Zn crystalline clusters. In other composition ranges, no XRD peak was detected, indicating that Zn_1−x_Te_x_ is amorphous, except for the highly Zn-rich composition. The spectroscopic ellipsometry results show the bandgap energy (*E*_*g*_) of the material as a function of composition (Fig. 2b). The *E*_*g*_ of pure Te was measured to be 0.6 eV. As the Zn content increases, *E*_*g*_ increases gradually, reaching the maximum value of 1.6 eV at Zn_0.35_Te_0.65_ and decreases again as the Zn content increases further. Figure 3c,d show X-ray photoelectron spectroscopy (XPS) spectra of pure Te, pure Zn, and Zn_0.35_Te_0.65_. Pure Te sample showed Te 3*d*_5/2_ peak at 572.85 eV, which is well known as the bulk Te peak. Similarly, pure Zn sample also showed Zn 2*p*_3/2_ peak at 1021.8 eV, which is well known as the bulk Zn peak. Binding energy shift measured in the XPS analysis indicates how the electronic states of elements in Zn_0.35_Te_0.65_ exist. All Zn 2*p*_3/2_ spectra detected from Zn_0.35_Te_0.65_ were Zn^2+^ peak at 1022.43 eV, while Te 3*d*_5/2_ spectra were sum of Te bulk peak at 572.85 eV and Te* peak at 573.18 eV. In other words, all the Zn atoms in the material are present in Zn^2+^ form having chemical bonding to Te atoms, while large proportion of Te remains as bulk Te and does not have a chemical bond with Zn, thus providing a large number of lone-pairs in the material. We could not find any reference for Te* peak, but as Te is known to have lots of oxidation states such as 6, 5, 4, 3, 2, 1, −1, −2, it is not strange to see the peak at 573.18 eV.

Figure 3 shows the electrical response of the Zn_1−x_Te_x_ devices and corresponding selector performances according to the material composition. The Zn_1−x_Te_x_ devices showed OTS behaviour in Te-rich composition (0.5 < x ≤ 1.0). Mixing Te with an appropriate amount of Zn (x ~ 0.65) dramatically improved the selector performance by reducing I_off_, but excessive Zn content (x > 0.65) caused deterioration of device performance and even metallic failure. The Zn-rich devices (0 < x < 0.5) exhibited high *off* state resistance in the low voltage region in the initial state, but became permanently metallic after external bias (~1 V) was applied. The electrical characteristics are closely related to those material properties shown above. The OTS behaviour observed from pure Te (Fig. 3a) and the large amount of unbound Te lone-pairs detected in the XPS spectra of the Te-rich Zn_1−x_Te_x_ device (Fig. 2c) show the correlation of Te atoms and OTS phenomenon. This is consistent with the previous theoretical studies suggesting that the sub-gap trap states originating from the lone-pairs of Te atoms trigger the OTS phenomenon^14–20^. The *off* state resistance of the OTS behaviour shown in the devices is strongly correlated with the *E*_*g*_ measured in the spectroscopic ellipsometry analysis. The *off* state resistance increases as *E*_*g*_ increases as a function of the composition change until it reaches the maximum value at Zn_0.35_Te_0.65_, and decreases again as *E*_*g*_ decreases with further compositional change. Pure Te alone cannot be used as selector application due to its high leakage current, while too much Zn may be a threat to the device. However, material composition optimization (Zn_0.35_Te_0.65_) makes the material suitable for selector application by modifying material parameters to suppress leakage currents effectively.

### Analytical modelling and performance-determining parameters

**Figure 4:**
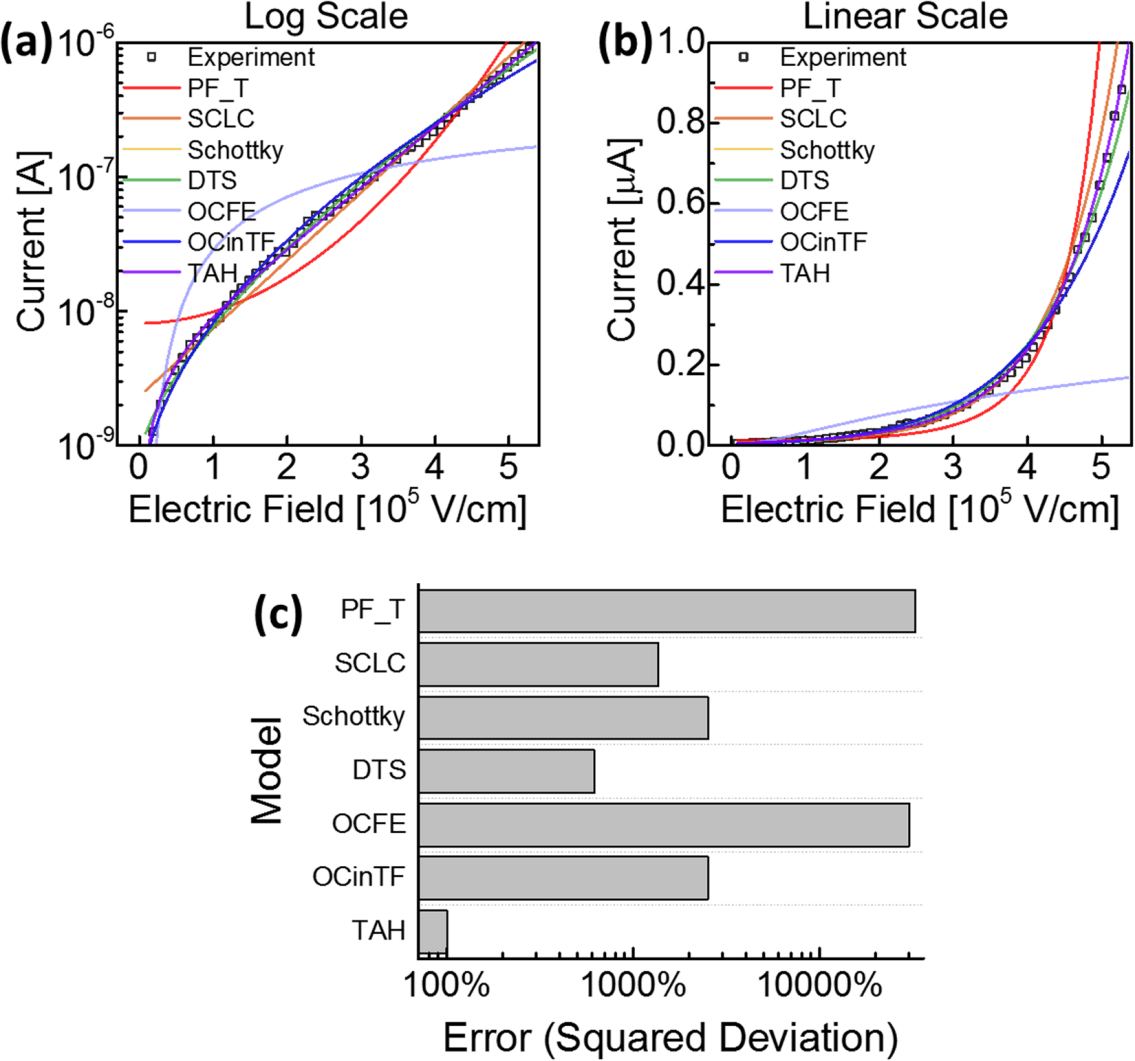
Comparison of the calculated results using various analytical models shown (**a**) in log scale and (**b**) in linear scale, and their (**c**) respective errors compared to the experimental data of Zn_0.35_Te_0.65_ OTS device. Among those models, thermally assisted hopping (TAH) provided remarkably small error compared to the others.

**Figure 5:**
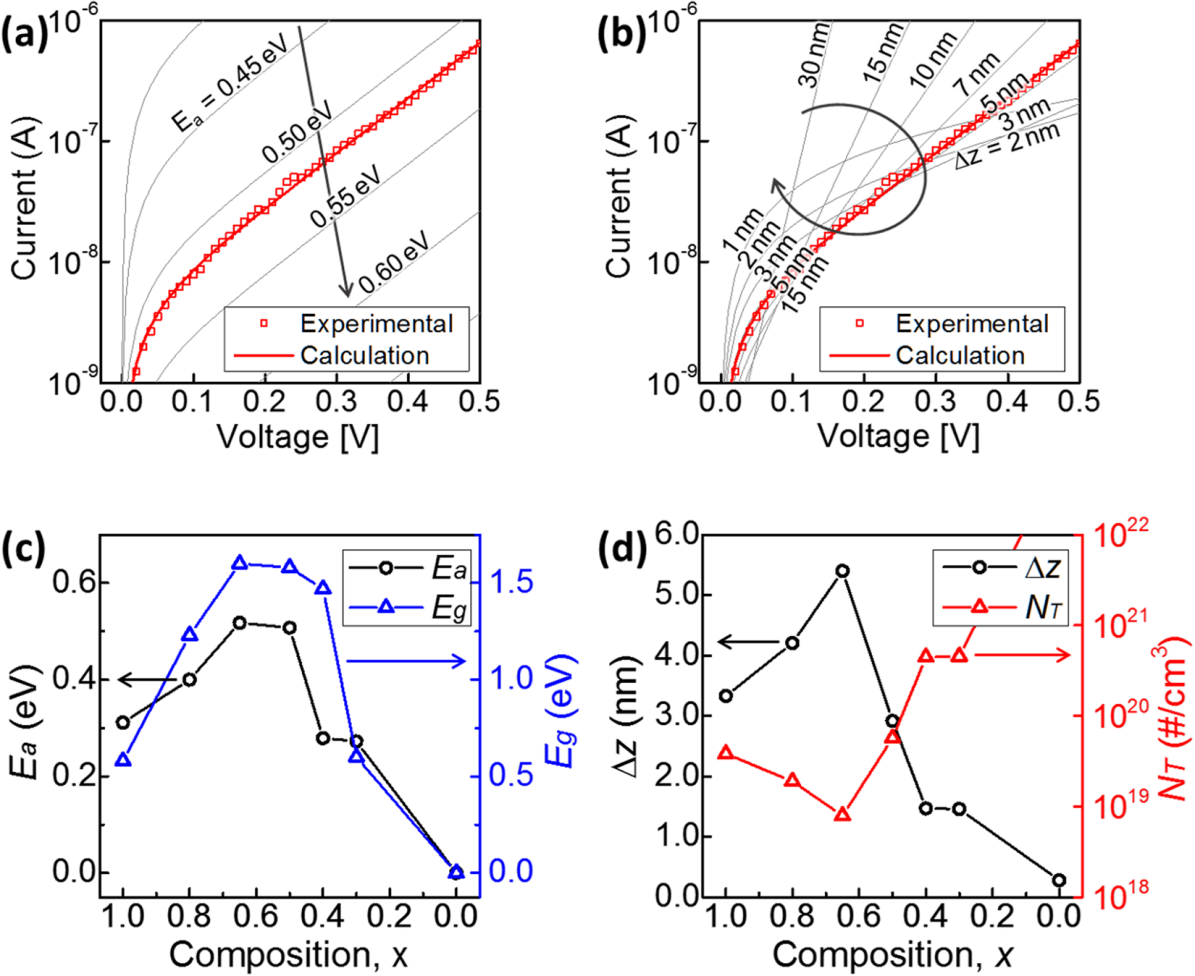
The performance-determining material parameters extracted from the analytical model. The calculated results are in good agreement with the experimental data ((**a**) *E*_*a*_ = 0.52 eV and (**b**) ∆z = 5.4 nm for the selector device with Zn_0.35_Te_0.65_), well explaining the remarkable leakage current inhibition in *off* state of the device. According to the model, higher *E*_*a*_ and moderate value of ∆z (3 nm < ∆z < 7 nm) maximizes the efficiency of leakage current inhibition. The material parameters extracted from the analytical model are in good agreement with the experimental observations. (**c**) The energy barrier *E*_*a*_ extracted from the electrical measurement by the analytical model and the band gap energy *E*_*g*_ optically measured by spectroscopic ellipsometry. (**d**) The average distance between deep traps ∆z and the density of deep traps responsible for the off state conduction *N*_*T*_. These material parameters well explains the electrical characteristics of the Zn_1−x_Te_x_ devices and their performance change depending on composition shown in Fig. 3a.

Analytical modelling on the OTS phenomenon has been widely developed in order to predict the electrical characteristics of the OTS devices. According to the previous literatures, candidate explanations for the conduction mechanism include Poole-Frenkel emission, Schottky emission, space-charge limited currents, optimum channel hopping, field-induced delocalization of tail states, percolation band conduction, transport through crystalline inclusions, and thermally assisted hopping^14,19,20^. The expressions for each model from previous literatures are summarized in Table 1. Figure 4 shows the prediction of each model and their respective errors compared to the experimental data of Zn_0.35_Te_0.65_ OTS device. Among those analytical models, thermally assisted hopping (TAH) model provided remarkably small error compared to the others. According to TAH model, the conduction in the OTS device could be understood by thermally assisted hopping conduction from localized traps to extended states, following eq. (1) ^14,19,20^:

1 $$I=2qA{N}_{T}\frac{{\rm{\Delta }}z}{{\tau }_{0}}exp(-\frac{{E}_{C}-{E}_{F}}{kT})\sinh (\frac{q{V}_{a}}{kT}\frac{{\rm{\Delta }}z}{2d})$$

where *q* is the elementary charge, *A* is the current path area, *N*_*T*_ is the density of deep traps responsible for the off state conduction, ∆*z* is the average distance between deep traps, *τ*_0_ is the attempt-to-escape time from a trapping site, (*E*_*C*_ − *E*_*F*_) is the energy barrier (*E*_*a*_) between the conduction band mobility edgy *E*_*C*_ and the quasi-Fermi level *E*_*F*_, and *d* is the thickness of the material. With the assumption of *τ*_0_ = 10^−15^ s, the results of the analytical model well explain the mechanism of the current inhibition of the material at low voltage as shown in Figs 4 and 5. According to the results, *E*_*a*_ and ∆*z* determines the off state conduction of the material. The magnitude of I_off_ is determined by *E*_*a*_, showing the magnitude of I_off_ reduced by 10 times for every 0.05 eV increase in *E*_*a*_ (Fig. 5a). Therefore, higher *E*_*a*_ is preferable for efficient leakage current inhibition. The dependency of I_off_ to the external electric field (in other words, the slope of the I–V curve) is determined by ∆z (Fig. 5b). Larger ∆z causes a steeper slope, and consequently higher I_off_ at 1/2 V_th_; while too small ∆z (<3 nm) causes higher I_off_ in the lower voltage region. Hence, a moderate value of ∆z (3 nm < ∆z < 7 nm) is preferable for selector application. *E*_*a*_ and ∆z according to the composition are shown in Fig. 5c and d. Correlation between the energy barrier *E*_*a*_ extracted from the analytical model and the band gap energy *E*_*g*_ measured by spectroscopic ellipsometry (Fig. 2b) shows the validity of the model (Fig. 5c). Both *E*_*a*_ and *E*_*g*_ have a maximum at a specific composition of x = 0.65, and gradually decrease when the composition reaches either end. ∆z showed similar dependency on the material composition and reached a maximum at x = 0.65 (Fig. 5d). The performance-determining parameters, *E*_*a*_ and ∆z, well explain the dependency of electrical properties on the material composition. In pure Te (x = 1), even though OTS phenomena occur, *E*_*a*_ and ∆z are both small, such that leakage current cannot be suppressed efficiently. Thus, pure Te is not sufficient to use as a selector device due to its high leakage current. As mixing Zn into Te, *E*_*a*_ and ∆z increases with Zn content until reaching their maximum at x = 0.65 composition. The leakage current was therefore minimized at x = 0.65, providing the best performance of the material for selector application. As the Zn content further increases excessively (0 < x < 0.5), *E*_*a*_ and ∆z decrease and thus the leakage current at low bias (<1 V) increases. Material composition optimization (Zn_0.35_Te_0.65_) makes the material suitable for selector application by modifying material parameters to suppress leakage current effectively. In other words, the key material parameters *E*_*a*_ and ∆z extracted from the model well explains how composition can maximize the selector device performances, by correlating the device performance to the material properties.

**Table 1:** Various analytical models and corresponding expressions on conduction of OTS materials from previous literatures.

Mechanism	Equation
Poole-Frenkel 1-center tunneling (PF1T)^22^	$\mathrm{ln}(I/I0)=\frac{\hslash {q}^{2}{F}^{2}}{3m}{(\frac{1}{kT}+\frac{1}{k{T}_{ph}})}^{2}$
Space-charge limited currents (SCLC)^22^	$\mathrm{ln}(I/I0)=\frac{\varepsilon F}{2\pi gqLkT}$
Schottky emission^22^	$\mathrm{ln}(I/I0)=\frac{1}{kT}\sqrt{\frac{{q}^{3}F}{\varepsilon }}$
Delocalization of tail states (DTS)^22^	$\mathrm{ln}(I/I0)={(\frac{\hslash qF}{\sqrt{m}})}^{2/3}(\frac{1}{kT}-\frac{1}{E0})$
Optimum channel field emission (OCFE)^22^	$\mathrm{ln}(I/I0)=-\,\sqrt{\frac{8\lambda {E}_{F}}{\alpha qF}}$
Optimum channel hopping, in thin film (OCinTF)^22^	$\mathrm{ln}(I/I0)=-\,\sqrt{\frac{8L\lambda }{\alpha }}+1.6\sqrt{\frac{qFL}{kT}}$
Thermally assisted hopping (TAH)^20^	$I=2qA{N}_{T}\frac{{\rm{\Delta }}z}{{\tau }_{0}}exp(-\frac{{E}_{C}-{E}_{F}}{kT})\sinh (\frac{q{V}_{a}}{kT}\frac{{\rm{\Delta }}z}{2d})$

The OTS phenomenon has been widely studied theoretically; however, its conduction mechanism and corresponding analytical model is still a highly controversial topic.

## Discussion

In summary, we have investigated the material characteristics and the device performance of Zn_1−x_Te_x_ binary OTS devices according to compositional change, using optical and electrical analysis methods. By comparing various analytical models, the electrical characteristics were best explained by an analytical model based on thermally assisted hopping conduction^14,19,20^, showing good agreement with the measured data. Consequently, the performance-determining material parameters were successfully extracted. Those parameters well explained the strong correlation between the material properties and the electrical device performance of Zn_1−x_Te_x_ in wide range of composition. The origin of OTS phenomenon and the mechanism of its conduction was explained experimentally and theoretically. Finally, the selector performance of the OTS device was successfully improved by optimizing the key parameters; the concentration of the sub-gap trap states and the bandgap energy of the material. This study provides better understanding of the sub-threshold conduction mechanism of the OTS device and gives clue for process optimization to maximize device performance of OTS selector devices.

## Methods

Zn_1−x_Te_x_ films of various compositions, including pure zinc (x = 0) and pure tellurium (x = 1), were deposited at room temperature using an RF magnetron sputtering system, in order to analyse their material characteristics and electrical properties. The material composition was measured using energy dispersive spectroscopy (EDS) analysis. For material property characterization, ZnTe films were deposited on flat wafers and were analysed by XRD, XPS and spectroscopic ellipsometry. For electrical property characterization, selector devices were fabricated with a W/ZnTe/W structure with 10 nm thick ZnTe layer. Tungsten was chosen as the electrode material, because of its advantages such as high electrical conductivity, high stability, and back-end-of-line (BEOL) compatibility. Device size was controlled by the size of W plug bottom electrode isolated by electrically insulating oxide sidewalls. The I–V response of the devices was measured using the following two measurement methods with a semiconductor device analyser using the circuit scheme shown in the inset of Fig. 1. Most of the electrical characteristics including the high resistance *off* state was measured in a longer time scale (>100 ms) with current compliance to prevent device damage due to the high current flow through the low *on* state resistance of the device. However, the *on* state conduction (shown in Fig. 1) was measured in a shorter time scale (<1 ms) in order to extract the intrinsic I_on_ of the device.
